# Overexpression of activated protein C hampers bacterial dissemination during pneumococcal pneumonia

**DOI:** 10.1186/s12879-014-0559-3

**Published:** 2014-11-04

**Authors:** Johannes Daan de Boer, Liesbeth M Kager, Joris JTH Roelofs, Joost CM Meijers, Onno J de Boer, Hartmut Weiler, Berend Isermann, Cornelis van ’t Veer, Tom van der Poll

**Affiliations:** Center for Infection and Immunity Amsterdam (CINIMA), Academic Medical Center, Amsterdam, The Netherlands; Center for Experimental and Molecular Medicine (CEMM), Academic Medical Center, Amsterdam, The Netherlands; Division of Internal Medicine, Academic Medical Center, Amsterdam, The Netherlands; Department of Pathology, Academic Medical Center, Amsterdam, The Netherlands; Department of Experimental Vascular Medicine, Academic Medical Center, Amsterdam, The Netherlands; Department Plasma Proteins, Sanquin, Amsterdam, The Netherlands; Blood Research Institute, Blood Center of Wisconsin, Milwaukee, WI USA; Department of Clinical Chemistry and Pathobiochemistry, Otto-von-Guericke University, Magdeburg, Germany; Center for Experimental and Molecular Medicine (CEMM), Center for Infection and Immunity Amsterdam (CINIMA), Academic Medical Center, University of Amsterdam, Meibergdreef 9, Room G2-130, Amsterdam, 1105 AZ The Netherlands

**Keywords:** Pneumonia, Inflammation, Coagulation, Activated protein C, Neutrophils

## Abstract

**Background:**

During pneumonia, inflammation and coagulation are activated as part of anti-bacterial host defense. Activated protein C (APC) has anticoagulant and anti-inflammatory properties and until recently was a registered drug for the treatment of severe sepsis. *Streptococcus (S.) pneumoniae* is the most common causative pathogen in community-acquired pneumonia.

**Methods:**

We aimed to investigate the effect of high APC levels during experimental pneumococcal pneumonia. Wild type (WT) and APC overexpressing (APC^high^)-mice were intranasally infected with *S. pneumoniae* and sacrificed after 6, 24 or 48 hours, or followed in a survival study.

**Results:**

In comparison to WT mice, APC^high^-mice showed decreased bacterial dissemination to liver and spleen, while no differences in bacterial loads were detected at the primary site of infection. Although no differences in the extent of lung histopathology were seen, APC^high^-mice showed a significantly decreased recruitment of neutrophils into lung tissue and bronchoalveolar lavage fluid. Activation of coagulation was not altered in APC^high^-mice. No differences in survival were observed between WT and APC^high^-mice (*P* =0.06).

**Conclusion:**

APC overexpression improves host defense during experimental pneumococcal pneumonia. This knowledge may add to a better understanding of the regulation of the inflammatory and procoagulant responses during severe Gram-positive pneumonia.

**Electronic supplementary material:**

The online version of this article (doi:10.1186/s12879-014-0559-3) contains supplementary material, which is available to authorized users.

## Background

The most common cause of community-acquired pneumonia (CAP) is infection with the pathogen *Streptococcus (S.) pneumoniae*[1],[2]. CAP often leads to severe sepsis and a recent study in sepsis patients showed that 35.6% of the cases were caused by severe CAP [3]. Pneumonia caused by *S. pneumoniae* and related pneumococcal sepsis are among the most common causes of death in the Western world rendering them a serious threat to health [4] and stressing the importance of understanding host defense mechanisms during this disease.

Over the last years many studies have demonstrated that the host response during severe (pneumococcal) pneumonia and sepsis consists of a strong pro-inflammatory response together with increased procoagulant activity, blunted anticoagulant mechanisms and suppression of the fibrinolytic system [2],[5],[6]. The interplay between inflammation and blood coagulation is considered to be an essential part of the host defense against infectious agents, a concept recently referred to as `immunothrombosis’ [7]. Activated protein C (APC) serves as one of the main inhibitors of the coagulation system via its capacity to inactivate coagulation factors Va and VIIIa. Additionally, in the last decade APC has gained much interest for its cytoprotective, anti-inflammatory, anti-apoptotic and vascular barrier-protective properties, which are largely mediated by the protease activated receptor-1 (PAR1) and the endothelial PC receptor (EPCR) [8],[9]. Much research has been done on the effect and role of APC during (pneumo)sepsis. In patients with severe pneumonia and sepsis, a correlation was observed between low PC and APC levels and the occurrence of organ dysfunction and adverse outcome, pointing to a protective role for APC [10]-[12]. Moreover, in the PROWESS study treatment of severe sepsis patients with intravenous recombinant human APC (rhAPC) reduced mortality [13] and patients with sepsis caused by *S. pneumoniae* pneumonia were among those with the largest benefit from rhAPC treatment [14],[15]. Despite these positive results, post-marketing studies failed to show similar protective effects of APC [16] and a recently completed confirmatory trial in septic shock patients did not show any benefit from APC treatment [17], leading to the withdrawal of this compound from the market [18].

Our laboratory recently showed protective effects of APC during pneumococcal pneumonia. Therapeutic administration of recombinant murine APC (rmAPC) attenuated pulmonary coagulopathy and improved survival during infection with *S. pneumoniae*[19], whereas early administration of rmAPC also had anti-inflammatory effects as reflected by reduced levels of pro-inflammatory cytokines in lungs of rmAPC-treated mice [20]. A possible limitation of these studies is the variability of rmAPC concentrations, due to its short half-live and the fact that in these studies rmAPC was administered only every 8 hours. To mimic the clinical situation more reliably, it would be better to administer rmAPC via continuous intravenous infusion. However, this is difficult to achieve in freely moving mice. Therefore, in the present study we used mice with sustained elevated levels of APC due to endogenous overexpression of hyperactivatable PC (APC^high^-mice [21]) and investigated the effects of elevated APC levels in the same model of pneumococcal pneumonia [19],[20]. We here show that sustained elevated levels of APC exert protective effects during pneumococcal pneumonia, as evidenced by decreased bacterial dissemination to distant body sites and decreased influx of inflammatory cells to the lungs, especially neutrophils.

## Methods

### Mice

Pathogen-free 10-week old female wild type (WT) C57BL/6 mice were purchased from Charles River (Maastricht, The Netherlands) and maintained in cages with 3–6 mice at the animal care facility of the Academic Medical Center (University of Amsterdam), according to national guidelines with free access to food and water. APC^high^-mice on a C57BL/6 genetic background were generated as described [21] and bred in the animal facility of the Academic Medical Center in Amsterdam. WT and APC^high^-mice were housed separately. Wellfare of the mice during the experiment was checked twice daily. As reported earlier, transgenic APC^high^ mice appear grossly normal, reproduce normally and do not show any signs of spontaneous bleeding [21]. Endogenous overexpression of APC in APC^high^ mice has been confirmed in previous reports [21] as well as in our own laboratory [22]. Per time-point two groups of 8 mice were compared (WT and APC^high^). The number of 8 mice per group was carefully chosen as with this number the Wilcoxon (Mann–Whitney) rank test gives a power of 80% and a chance of 0.904 that the observed mean in one group is lower than in the other group, demonstrated with a two-sided level of significance of 0.05. Moreover, this group size provided us statistical significance for differences we consider clinically relevant.

### Ethics statement

Mice studies were carried out under the guidance of the Animal Research Institute of the Academical Medical Center in Amsterdam (ARIA). All animals were maintained at the animal care facility of the Academic Medical Center (University of Amsterdam), with free access to food and water, according to National Guidelines for the Care and Use of Laboratory Animals, which are based on the National Experiments on Animals Act (Wet op de Dierproeven (WOD)) and the Experiments on Animals Decree (Dierproevenbesluit), under the jurisdiction of the Ministry of Public Health, Welfare and Sports, the Netherlands. The Committee of Animal Care and Use (Dier Experimenten Commissie, DEC) of the University of Amsterdam approved all experiments (Permit number DIX100121-101787).

### Experimental infection and determination of bacterial growth

Pneumonia was induced by intranasal inoculation of all mice at the same time point of 5*10^4^ colony forming units (CFU) in 50 μl 0.9% NaCl of *S. pneumoniae* serotype 3 (ATCC strain 6303; American Type Culture Collection, Rockville, MD) as previously described [19],[20]. Mice were sacrificed after 6, 24 or 48 hours (*n* = 8/group) and survival studies (*n* =16/group for WT mice and 12/group for APC^high^-mice) were performed in which mortality was assessed every 6 hours. Sample harvesting, processing and determination of bacterial growth were done as described [19],[20]. Briefly, mice were sacrificed under intraperitoneal anesthesia containing ketamin (Eurovet Animal Health, Bladel, The Netherlands) and medetomidin (Pfizer Animal Health Care, Capelle aan den IJssel, The Netherlands). Blood was drawn from the vena cava inferior into syringes containing sodium citrate (4:1 vol/vol) and stored on ice. Left lungs, spleen and liver were harvested, weighed and homogenized at 4°C in 4 volumes of sterile saline using a tissue homogenizer (Pro200, Pro Scientific Inc., Oxford, CT) and processed as described below. CFU were determined from serial dilutions of organ homogenates and blood plated on blood agar plates and incubated at 37°C 5% CO_2_ for 20 h before colonies were counted. Bronchoalveolar lavage fluid (BALF) was harvested by selectively cannulation of the right main bronchus with a 22-gauge Abbocath-T catheter (Abott, Sligo, Ireland) followed by unilateral lavage of the right lung with three 300 μl aliquots of sterile phosphate-buffered saline. Harvested cells were kept on ice until analysis.

### Assays

Lung homogenates were diluted 1:2 in lysis buffer containing 300 mM NaCl, 30 mM Tris, 2 mM MgCl_2_, 2 mM CaCl_2_, 1% Triton X-100 and protease inhibitor cocktail (Roche, Indianapolis, IN) and incubated at 4°C for 30 min and centrifuged at 680 *g* at 4°C for 10 min. Lung homogenates were stored at −20°C until analysis. Blood was centrifuged at 600 *g* and plasma was snap frozen in liquid nitrogen and stored at −80°C until analysis. Interleukin (IL)-6, IL-10, IL-12p70, interferon (IFN)-γ and monocyte-chemoattractant protein (MCP)-1 were measured by cytometric bead array (CBA) multiplex assay (BD Biosciences, San Jose, CA) in accordance with the manufacturers’ recommendations. Tumor necrosis factor-α (TNF-α; R&D systems, Minneapolis, MN), keratinocyte-derived chemokine (KC; R&D systems, Minneapolis, MN) and thrombin-antithrombin complexes (TATc; Siemens Healthcare Diagnostics, Marburg, Germany) were measured with commercially available ELISA kits according to the manufacturers’ instructions.

### Histology and immunohistochemistry

Paraffin-embedded 4 μm lung sections were stained with haematoxylin and eosin (H&E) and analyzed for inflammation and tissue damage, as described previously [19],[20]. All slides were scored by an experienced histopathologist blinded for experimental groups for the following parameters: bronchitis, interstitial inflammation, edema, endothelialitis, pleuritis and thrombus formation. All parameters were rated separately from 0 (condition absent) to 4 (most severe condition). The total histopathology score was expressed as the sum of the scores of the individual parameters, with a maximum of 24. Staining for granulocytes, using fluorescein isothiocyanate-labeled rat-anti-mouse Ly-6G mAb (BD Pharmingen, San Diego, CA) was performed as described previously [22],[23]. All slides were slightly counterstained with methylgreen. The total tissue area of Ly-6G stained slides was scanned with a slide scanner (Olympus dotSlide, Tokyo, Japan) and the obtained scans were exported in TIFF format for digital image analysis. The digital images were analyzed with ImageJ (version 2006.02.01, National Institutes of Health, Bethesda, MD) and the immunopositive (Ly6G+) area calculated from an average of 10 images per lung was expressed as the percentage of the total lung surface area.

### Cell counts and differentials

Freshly harvested BALF-cells were counted using an automatic haemocytometer (Beckman Coulter, Fullerton, CA). Differential counts were performed on cytospins stained with a modified Giemsa stain (Diff-Quick, Dade Behring, Düdingen, Switzerland).

### Statistical analysis

All data are expressed as box and whisker plots showing the smallest observation, lower quartile, median, upper quartile and largest observation. Comparisons between groups were performed using a Mann–Whitney *U* test. For survival studies Kaplan-Meier analyses followed by log rank test were performed. All analyses were done using GraphPad Prism version 5.01 (GraphPad Software, San Diego, CA). *P*-values <0.05 were considered statistically significant.

## Results

### APC-overexpression hampers bacterial dissemination

**Figure 1:**
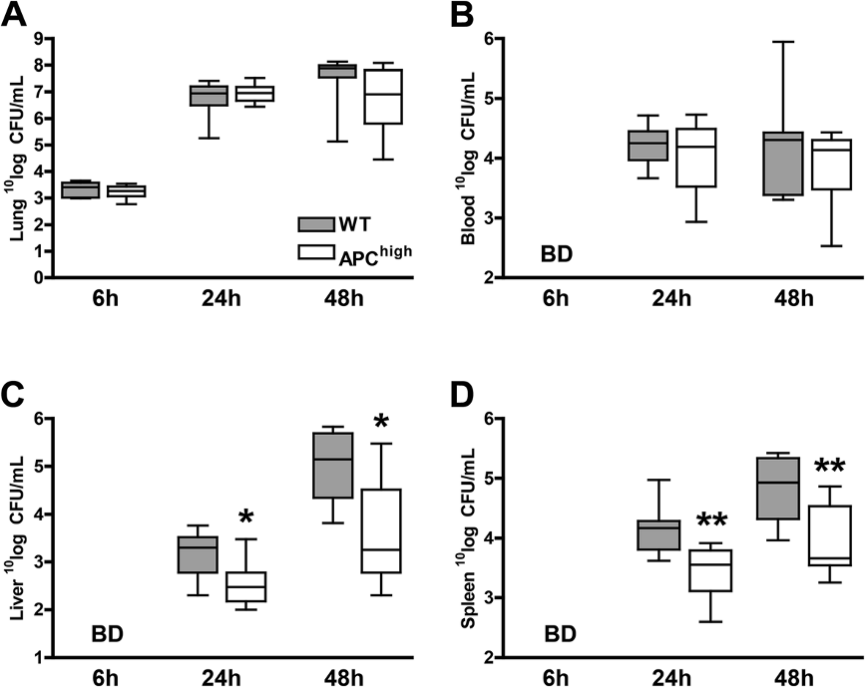
**APC-overexpression is associated with reduced bacterial dissemination** ***.*** Mice were intranasally inoculated with 5*10^4^ CFU of *S. pneumoniae* and sacrificed after 6, 24 or 48 hours. Bacterial loads were determined in lung homogenates **(A)**, blood **(B)**, liver homogenates **(C)** and spleen homogenates **(D)**. Data are expressed as box and whisker plots showing the smallest observation, lower quartile, median, upper quartile and largest observation. Grey boxes represent WT mice, white boxes represent APC^high^-mice (*n* =8 mice per group for each time point). *P <0.05 and **P <0.01 for the difference between WT and APC^high^-mice (Mann–Whitney *U* test). BD below detection limits.

We previously showed that APC^high^-mice have markedly increased levels of APC in various organs as was measured in plasma, lung-, kidney-, liver-, and spleen homogenates of uninfected APC^high^-mice [22]. To investigate whether overexpression of APC would impact on bacterial growth, we infected WT and APC^high^-mice with 5*10^4^ CFU of *S. pneumoniae* and sacrificed them after 6, 24 or 48 hours to determine bacterial loads in lungs (the primary site of infection), blood, liver and spleen homogenates (to evaluate the extent of dissemination) (Figure 1). No differences in bacterial growth were seen in the lungs (Figure 1A) or blood (Figure 1B) between WT and APC^high^-mice. However, relative to WT mice, APC^high^-mice showed markedly decreased bacterial loads 24 and 48 hours after infection in both liver (Figure 1C; *P* <0.05 for both time points) and spleen homogenates (Figure 1D; *P* <0.01 for both time points). These data indicate that overexpression of APC hampers bacterial dissemination during *S. pneumoniae*-induced pneumonia.

### APC-overexpression does not impact on lung pathology during pneumococcal pneumonia but is associated with decreased neutrophil influx

**Figure 2:**
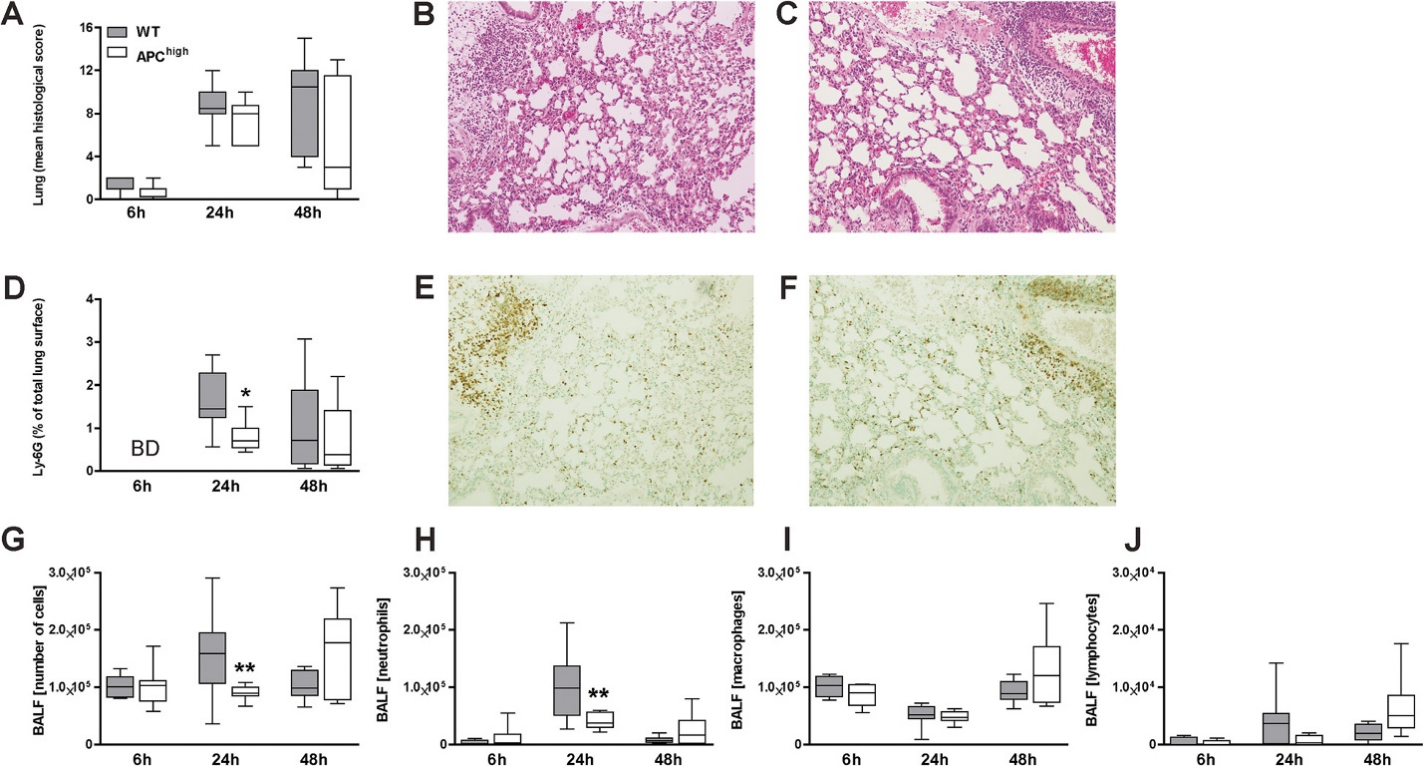
**APC-overexpression does not impact on lung pathology but attenuates neutrophil influx into the lungs** ***.*** Histology scores were similar in WT and APC^high^-mice 6, 24 and 48 hours post-infection with 5*10^4^ CFU of *S. pneumoniae* **(A)**. Representative photographs of WT **(B)** and APC^high^-mice **(C)** (H&E staining, original magnification x100) 48 hours post-infection. Decreased granulocyte influx as measured by Ly-6G expression in APC^high^-mice infected with *S. pneumoniae,* 24 hours after infection **(D)**. Representative photographs of Ly-6G immunostaining (original magnification x100) for granulocytes of WT **(E)** and APC^high^-mice **(F)**, 24 hours post-infection. APC^high^-mice showed decreased numbers of total cell counts in bronchoalveolar lavage fluid (BALF) compared to WT mice **(G)**, which was caused by a decreased influx of neutrophils **(H)**. No differences in influx of macrophages **(I)** and lymphocytes **(J)** were found. Data are expressed as box and whisker plots showing the smallest observation, lower quartile, median, upper quartile and largest observation. Numbers of Ly-6G + granulocytes are expressed as the percentage of the total lung surface area. Neutrophils, macrophages and lymphocytes are expressed as absolute numbers. Grey boxes represent WT mice, white boxes represent APC^high^-mice (*n* =8 mice per group for each time point). *P <0.05 and **P <0.01 for WT versus APC^high^-mice (Mann–Whitney *U* test).

Our model of pneumococcal pneumonia is associated with profound lung pathology [19],[20],[24]. Both WT and APC^high^-mice infected with *S. pneumoniae* showed inflammatory infiltrates in the lungs characterized by bronchitis, interstitial inflammation, oedema, endothelialitis, pleuritis and thrombus formation (Figure 2A-C). However, APC-overexpression did not induce differences in lung pathology during infection between WT and APC^high^-mice (Figure 2A). Neutrophils contribute to host defense against *S. pneumoniae*[19],[20]. In order to investigate neutrophil influx in our model we measured percentage of Ly-6G staining in infected lung tissue (Figure 2D-F). Twenty-four hours after infection, APC^high^-mice displayed significantly decreased neutrophil numbers in their lungs as compared to WT mice (*P* <0.05; Figure 2D-F). These data were confirmed by decreased numbers of total cell counts in BALF in APC^high^-mice compared to WT mice (*P* <0.01; Figure 2G), for which a decrease in the number of neutrophils in APC^high^-mice mainly was responsible (*P* <0.01; Figure 2H). BALF macrophage and lymphocyte numbers did not differ between APC^high^ and WT mice (Figure 2I-J).

### Limited differences between WT and APC^high^-mice in cytokine production

Since cytokines are important regulators of the host immune response during pneumococcal pneumonia [1],[2] we measured levels of the following cytokines in lung homogenates and plasma: TNF-α, IL-6, IL-10, IL-12p70, IFN-γ, MCP-1 and KC (Table 1). The lung and plasma levels of these mediators did not differ between mouse strains at 6, 24 or 48 hours after infection.

**Table 1:** **Cytokine concentrations in lung homogenates and plasma of WT and APC** ^**high**^ **-mice during pneumococcal pneumonia**

	Lung homogenates					
*all [pg/ml]*	WT	APC^high^	WT	APC^high^	WT	APC^high^
	T = 6		T = 24		T = 48	
**TNF-α**	8.6	7.6	249	284	217	56
	(6.1-9.8)	(4.8-14)	(164–318)	(182–549)	(19–821)	(11–290)
**IL-6**	21	31	1218	1429	1317	694
	(17–22)	(16–51)	(474–1522)	(1078–1992)	(650–1722)	(104–1449)
**IL-10**	16	24	17	12	20	19
	(15–22)	(22–35)	(11–24)	(7.6-21)	(11–33)	(16–28)
**IL-12p70**	BD	BD	13	17	BD	BD
			(5.4-21)	(11–49)		
**IFN-γ**	BD	BD	12	19	14	13
			(8.1-19)	(8.4-26)	(4.3-32)	(5.4-41)
**MCP-1**	254	210	2891	3239	4913	3056
	(229–274)	(187–247)	(1935–3561)	(2507–4730)	(2901–5574)	(884–5958)
**KC**	255	221	4809	6838	8338	6993
	(215–317)	(66–347)	(3548–7702)	(5165–9143)	(4703–10317)	(4421–8496)
**KC (BALF)**	117	102	255	277	231	236
	(106–131)	(99–127)	(241–347)	(252–306)	(213–343)	(194–260)
	**Plasma**					
	**WT**	**APC** ^**high**^	**WT**	**APC** ^**high**^	**WT**	**APC** ^**high**^
	**T = 6**		**T = 24**		**T = 48**	
**TNF-α**	15	16	42	37	92	43
	(13–20)	(13–18)	(36–53)	(29–46)	(48–111)	(23–74)
**IL-6**	5.1	3.9	331	263	795	223
	(2.5-7.4)	(2.9-22)	(232–442)	(213–354)	(183–881)	(111–602)
**IL-10**	BD	BD	BD	BD	BD	BD
**IL-12p70**	7.8	10	11	27	6.9	6.0
	(5.2-9.8)	(6.4-13)	(10–16)	(14–40)	(3.5-17)	(3.9-8.4)
**IFN-γ**	2.0	1.4	24	38	43	29
	(1.9-2.1)	(1.3-6.7)	(14–41)	(23–55)	(30–69)	(8.2-82)
**MCP-1**	26	37	138	95	590	240
	(26–41)	(35–42)	(71–196)	(83–149)	(402–786)	(138–786)

Lung and plasma cytokine levels after intranasal infection with 5*10^5^ CFU of *S. pneumoniae*. Wild type (WT) and APC^high^-mice were sacrificed 6, 24 or 48 hours after infection. Data are expressed as median (interquartile range) of *n* =8 mice per group per time point. BALF broncho-alveolar lavage fluid, BD below detection limits, TNF-α tumor necrosis factor-α, IL interleukin, IFN-γ interferon-γ, KC keratinocyte-derived chemokine, MCP-1 monocyte chemotactic protein.

### Overexpression of APC does not inhibit coagulation activation

**Figure 3:**
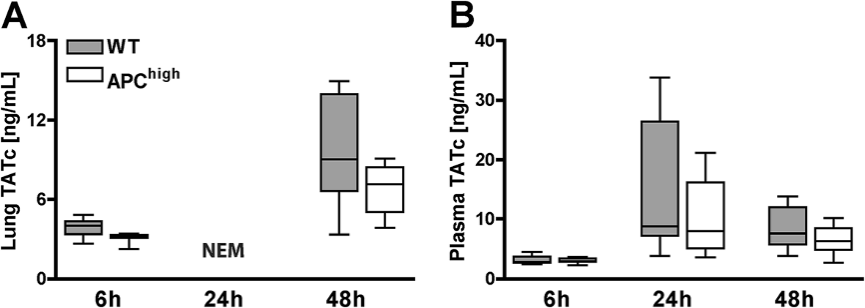
**APC** ^**high**^ **-mice show an unaltered procoagulant response.** Mice were infected intranasally with 5*10^4^ CFU of *S. pneumoniae*. After 6 and 48 hours levels of TATc were measured in lung homogenates **(A)** and plasma **(B)** of WT and APC^high^-mice. No differences were seen between WT and APC^high^-mice. Grey boxes represent WT mice, white boxes represent APC^high^-mice (*n* =8 mice per group for each time point). *P <0.05 for WT versus APC^high^-mice (Mann–Whitney *U* test). NEM not enough material for analysis.

To obtain insight into the anticoagulant potential of overexpression of APC we measured the levels of TATc, a parameter of coagulation-induction. TATc was measured in in lung homogenates and plasma of WT and APC^high^-mice 6 and 48 hours after inoculation with *S. pneumoniae* (Figure 3). No differences in TATc levels between WT and APC^high^-mice were observed either in the pulmonary or the systemic compartment.

### APC^high^-mice show a trend towards a better survival during experimental pneumococcal pneumonia

**Figure 4:**
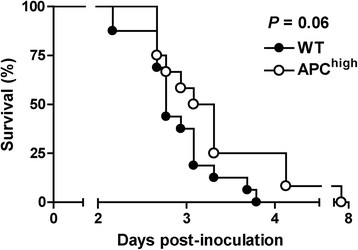
**Survival.** APC^high^-mice show a trend towards a delayed mortality during pneumococcal pneumonia. Wild type (WT) (*n* =16) and APC^high^-mice (*n* =12) were infected intranasally with 5*10^4^ CFU of *S. pneumoniae* and mortality was assessed every 6 hours, Comparison between groups was done by using Kaplan-Meier analysis followed by log rank tests.

We have previously shown that in our model of pneumococcal pneumonia is associated with a high mortality [19]. To investigate whether overexpression of APC impacts mortality during pneumococcal pneumonia we intranasally infected WT and APC^high^-mice with 5*10^4^ CFU of *S. pneumoniae* and observed them during the following 8 days (Figure 4). In line with the decreased bacterial burdens observed in liver and spleen homogenates, and the less prominent cellular influx in the lung, APC^high^-mice showed a trend towards a better survival when compared to WT mice (*P =*0.06).

## Discussion

The potential of the PC system as a pharmaceutical target in sepsis remains a topic of debate despite the retraction of recombinant human APC from the market due to efficacy concerns [13],[17],[18],[25]-[27]. In the pulmonary compartment, increased coagulation activation during pneumonia may be advantageous for the host by preventing potential lethal bleeding from destructed lung tissue and by providing a platform to encounter, entrap and kill invading pathogens [7]. In contrast, inflammation-induced coagulation activation can also produce collateral damage and be detrimental by interfering with normal organ physiology and hampering a balanced immune response [5],[6],[28]. APC has both anticoagulant and anti-inflammatory effects [8],[9] and thereby can be expected to influence the host response during infection [8],[9]. Our goal was to address the impact of sustained elevated levels of APC in a clinically relevant pneumonia model by using APC^high^-mice with high endogenous hyperactivatable PC expression [21].

The results of our study show that in *S. pneumoniae-*induced pneumonia APC^high^-mice have an improved host defense as reflected by decreased dissemination of bacteria to distant organs and an attenuated influx of neutrophils into the lungs. In the `PROWESS’ human sepsis trial, rhAPC treatment was especially beneficial in the sepsis group with pneumonia [13],[14]. Therefore, we focused this and our previous studies [19],[20] on effects of APC in the pulmonary compartment and CAP, with *S. pneumoniae* as the clinically most relevant pathogen [1],[2]. The APC^high^-mice used in this study overexpress hyperactivatable human PC [21]. In this respect, generation of APC is dependent on thrombin, yet less dependent on thrombomodulin (which is downregulated in sepsis), thereby conferring some “clot-specificity”. Therefore, overexpression in APC^high^-mice is different from infusing recombinant APC per se. Nevertheless, because there is constitutive thrombin generation, the animals show constitutively elevated APC at a concentration that is very similar to the dosing used in clinical trials (45 ng/ml steady state) [29].

Likewise, inhibition of neutrophil attraction by APC was shown in other human and animal studies in different inflammatory settings [30]-[34]. Treatment with rhAPC in healthy volunteers challenged with lipopolysaccharide (LPS) by bronchoscopy resulted in decreased neutrophil migration [35]. In a nice series of experiments involving both in vivo (LPS challenge in mice) and in vitro experiments (neutrophil agarose migration assay) APC was shown to desensitize neutrophils for chemotaxis in an integrin-dependent manner, without affecting neutrophil function, cytokine production or apoptosis [30]. Here, we observed a similar specific inhibitory effect of APC affecting the influx of neutrophils when using live bacteria instead of LPS. This effect of APC was observed at 24 hours after inoculation of *S. pneumoniae*, while at the 48-hour time point neutrophil numbers in lungs and KC levels in lung homogenates and BALF were similar in both mouse strains, suggesting that during fulminant pneumonia the inhibitory effect of APC on neutrophil recruitment is overruled by the strong proinflammatory stimulus provided by high bacterial loads. We did not observe differences in cytokine levels between APC^high^ and WT mice, suggestive of similar chemotactic gradients. In accordance, APC has been shown to inhibit IL-8 directed [33] and integrin-mediated neutrophil migration [30]. In addition, a recent study showed that APC might control inflammation via leukocyte adhesion in a RAGE-dependent way [36]. Of note, the decreased amount of pulmonary neutrophils did not result in increased local CFU counts, suggesting that the number of neutrophils was still adequate to cope with the pneumococci in the lungs.

Although bacterial loads were comparable at the primary site of infection, APC^high^-mice had lower burdens of pneumococci in liver and spleen. The endothelium is the anatomical barrier that pathogens need to cross before dissemination from the lungs to the systemic compartment and distant organs. The integrity of the endothelium is tightly regulated, in which sphingosin-1-phosphatase (S1P) plays an important part [37]. APC is able to exert S1P agonistic effects and thereby to improve endothelial barrier function [38], which may in part explain the reduced dissemination of *S. pneumoniae* from the lungs of APC^high^-mice. Of note, APC^high^-mice had higher bacterial loads in liver and spleen when compared with WT mice in spite of similar bacterial counts in blood. Conceivable explanations for this discrepancy include the possibility that the small amount of blood plated for culture (50 μl) yields less representative results when compared with whole organ homogenates, due to intermittent bacterial spillage to the circulation from different organs, and/or the option that high APC levels have more profound effects on bacterial growth in the cellular environment of intact organs such as the liver and spleen. Clearly, high APC levels primarily affected bacterial dissemination, considering that APC^high^ and WT mice had similar bacterial burdens at the primary site of infection.

Remarkably, APC^high^-mice did not show attenuated coagulation compared to WT mice. In our previous studies using this model of pneumococcal pneumonia administration of rmAPC did exert anticoagulant effects [19],[20]. Notably, the high intraperitoneal APC doses (125 μg every 8 hours) used in these earlier investigations resulted in APC levels one hour post-injection that were almost 10-fold higher than the APC levels measured in APC^high^-mice [19],[20],[22]. In addition, the APC expressed in APC^high^-mice is described to have 35% less anticoagulant effect *in vitro*. Together, these facts may at least in part explain the lack of a clear anticoagulant effect in APC^high^-mice with pneumococcal pneumonia. Nonetheless, in spite of this APC^high^-mice displayed a clear protective phenotype, suggesting that the cytoprotective effects of APC were mainly responsible here for.

The current results contrast with our recent investigation in which we studied APC^high^-mice in severe Gram-negative pneumonia derived sepsis caused by *Burkholderia pseudomallei*, the causative agent of melioidosis [22]. APC^high^-mice demonstrated enhanced susceptibility to *B. pseudomallei* infection compared with WT mice as indicated by a strongly increased mortality accompanied by enhanced bacterial loads in the lungs, blood, and distant organs 48 hours after infection [22]. Although a clear explanation for these different results is lacking, clearly, the models and pathogens used differ considerably, which is very well documented in a wide variety of experimental settings. In fact, this is an interesting point which may, if true in the human situation, might in part explain the overall poor efficacy of rhAPC in sepsis trials.

Our study is limited by the fact that we studied only one infectious dose of a serotype 3 pneumococcus. In the limited experience we have with lower doses of this highly virulent bacterial strain the onset of disease was somewhat postponed without clear differences in the eventual outcome. Nonetheless, further studies are required to establish the phenotype of APC^high^-mice after infection with other doses of the *S. pneumoniae* strain used here, and with other pneumococcal serotypes. In addition, APC^high^-mice do not adequately mimic the clinical scenario of continuous intravenous APC infusion started at the time of already established infection, a setting difficult to accomplish in freely moving mice. As such, our data cannot be directly extrapolated to the effect of high APC levels achieved at various durations after induction of pneumonia. The current results, obtained with mice exposed to sustained high APC levels, confirm and extend our earlier results obtained with repeated administration of bolus doses of recombinant APC during pneumococcal pneumonia, resulting in a more artificial situation of highly variable APC concentrations due to the short half-life of this protein [19],[20].

## Conclusions

In summary, this study shows that elevated formation of human APC improves host defense during experimental pneumococcal pneumonia with respect to reduced bacterial dissemination and mitigated neutrophil influx in the lungs. Although recombinant APC has been withdrawn from the market these results support previous preclinical data showing beneficial effects of APC in experimental models of sepsis and pneumonia.

## Authors’ original submitted files for images

Below are the links to the authors’ original submitted files for images. Authors’ original file for figure 1 Authors’ original file for figure 2 Authors’ original file for figure 3 Authors’ original file for figure 4

## Abbreviations

APC: Activated protein C
BALF: Bronchoalveolar lavage fluid
CAP: Community-acquired pneumonia
CBA: Cytometric bead array multiplex assay
CFU: Colony forming units
EPCR: Endothelial protein C receptor
H&E: Haematoxylin and eosin
IFN-γ: Interferon-γ
IL: Interleukin
KC: Keratinocyte-derived chemokine
LPS: Lipopolysaccharide
MCP-1: Monocyte-chemoattractant protein-1
PAR-1: Protease activated receptor-1
PC: Protein C
rmAPC: recombinant murine activated protein C
rhAPC: recombinant human activated protein C
S1P: Sphingosin-1-phosphatase
*S. pneumoniae*: *Streptococcus pneumonia*
TATc: Thrombin-antithrombin complexes
TNF-α: Tumor necrosis factor-α
WT: Wild type
