# Supplementary figures and images for: Overexpression of activated protein C hampers bacterial dissemination during pneumococcal pneumonia
Source: BMC Infect Dis. 2014 Nov 4;14:559. doi: 10.1186/s12879-014-0559-3 (PMC4228088; doi:10.1186/s12879-014-0559-3)

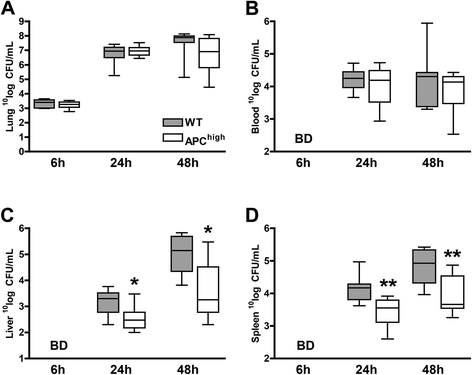

Supplement: Supplementary file 1 — Authors’ original file for figure 1 [file 12879_2014_559_MOESM1_ESM.gif]

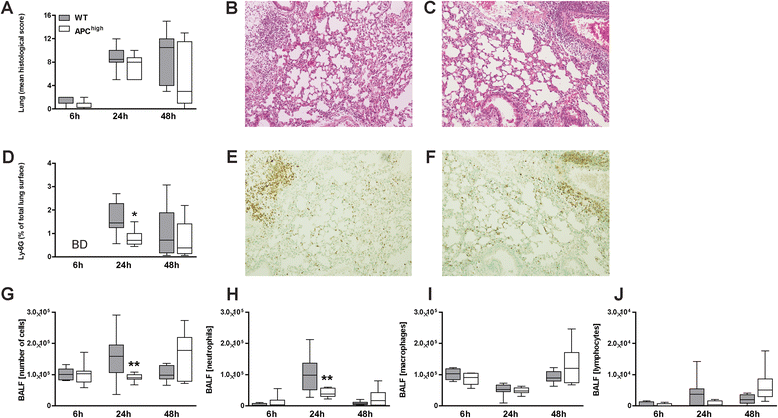

Supplement: Supplementary file 2 — Authors’ original file for figure 2 [file 12879_2014_559_MOESM2_ESM.gif]

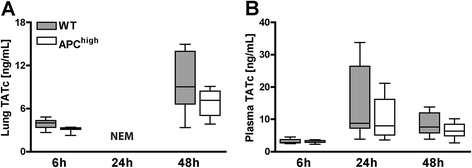

Supplement: Supplementary file 3 — Authors’ original file for figure 3 [file 12879_2014_559_MOESM3_ESM.gif]

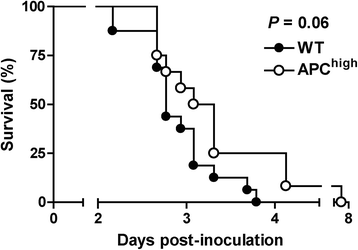

Supplement: Supplementary file 4 — Authors’ original file for figure 4 [file 12879_2014_559_MOESM4_ESM.gif]
